# Supplementary material for: Acceptance and compliance with micronutrient powder (MNP) among children aged 6–23 months in northern Nigeria
Source: PLOS Glob Public Health. 2022 Oct 17;2(10):e0000961. doi: 10.1371/journal.pgph.0000961 (PMC10022258; doi:10.1371/journal.pgph.0000961)
Supplement: S1 Dataset — (ZIP) [file pgph.0000961.s008.zip › S8_Dataset/Spot Checks.zip!/Spot Checks/O7O72015_A_GA_PM_2_SC.pdf]

# SPOT CHECK RECORDING FORM

**State: __ADAMAWA______________ LGA: _GANYE________________ Community: _SANSIRI____________________**

**Sex of index child: _F_____________ Age of index child (in months): _7_________________**

## Spot Check Information

1. **Date of MNP distribution to household** (circle option below based on LGA name above)**:**
   - **Kebbi:** BirninKebbi&Jega LGA: **June 16**
   - **Kebbi:** Danko-wasagu: **June 17**
   - **Adamawa:**Song, Ganye: (June 18)
   - **Adamawa:**Guyuk **June 19**
2. **Date of Spot Check Observation: _6-07-2015__________________**
3. **Number of sachets remaining**(Ask the caregiver to show you and count the specific number of sachets remaining)**:**

   43

4. **Number of sachets that should remainusing one MNP sachet per day** (Analyst only)**:**

   43

5. **Number of sachets remaining / number of sachets should be remaining**(Analyst only)**:**

   **______43____ / ___43______ = __100________ %**

## Probes

**Question 1.** You have **XX** MNP sachets remaining. Explain how easy or difficult it was to use the MNP product on a daily basis for your child 6 – 23 months? (Circle one answer)

**Very Difficult** **Difficult** **Easy** **(Very Easy)**

**Question 2.**Describe any specific challenges that you faced that made the MNP dif**(**ficult to use on a daily basis?THERE WAS NO CHALLENGE

**Question 3.** Describe specific aspects of the MNP product that you or your child enjoyed that made it easier to use on a daily basis?THE MNP MAKES THE CHILD TO SLEEP VER WELL FOR A PROLONG HOURS ON A DAILY BASIS…..THE MNP HAS INCREASED CONSIDERABLY THE AMOUNT OF FOOD INTAKE BY THE CHILD ON A DAILY BASIS…… THE CHID HAS ADDED A BIT OF WEIGHT…….THE BABY'S FATHER IS REALLY INTRESTED IN ENSURING THAT THE BABY TAKES THE MNP REGULARLY.

**Question 4.** What questions do you have about the MNP based on your experience using it? WHAT IS THE MEANING OF MIXME/……….DOES THE MNP HELPS DEVELOP THE BRAIN?.........CAN THE MNP BE GIVEN WITH OTHER DRUGS IN CASE THE CHILD IS SICK AND ON MEDICATIONS?............WHAT HAPPEN IF I FORGET TO GIVE MY CHILD THE MNP FOR LIKE 3DAYS.

**Question 5.** What specific recommendations do you have that we can do as a program to make the MNP easier to use on a daily basis?
